# The Interactions of DNA Repair, Telomere Homeostasis, and p53 Mutational Status in Solid Cancers: Risk, Prognosis, and Prediction

**DOI:** 10.3390/cancers13030479

**Published:** 2021-01-27

**Authors:** Pavel Vodicka, Ladislav Andera, Alena Opattova, Ludmila Vodickova

**Affiliations:** 1 Institute of Experimental Medicine, AS CR, 142 20 Prague, Czech Republic; pavel.vodicka@iem.cas.cz (P.V.); alena.opattova@iem.cas.cz (A.O.); 2 First Medical Faculty, Charles University, 121 08 Prague, Czech Republic; 3 Biomedical Center, Faculty of Medicine in Pilsen, Charles University, 301 00 Pilsen, Czech Republic; 4 Institute of Biotechnology, AS CR, 252 50 Vestec, Czech Republic; ladislav.andera@ibt.cas.cz; 5 Institute of Molecular Genetics, AS CR, 142 20 Prague, Czech Republic

**Keywords:** interactions, DNA damage response, telomere homeostasis, *TP53* mutational status, cancer risk, cancer progression, cancer therapy

## Abstract

**Simple Summary:**

p53 is a nuclear transcription factor with a pro-apoptotic function. Somatic mutations in this gene represent one of the most critical events in human carcinogenesis. The disruption of genomic integrity due to the accumulation of various kinds of DNA damage, deficient DNA repair capacity, and alteration of telomere homeostasis constitute the hallmarks of malignant diseases. The main aim of our review was to accentuate a complex comprehension of the interactions between fundamental players in carcinogenesis of solid malignancies such as DNA damage response, telomere homeostasis and *TP53*.

**Abstract:**

The disruption of genomic integrity due to the accumulation of various kinds of DNA damage, deficient DNA repair capacity, and telomere shortening constitute the hallmarks of malignant diseases. DNA damage response (DDR) is a signaling network to process DNA damage with importance for both cancer development and chemotherapy outcome. DDR represents the complex events that detect DNA lesions and activate signaling networks (cell cycle checkpoint induction, DNA repair, and induction of cell death). *TP53*, the guardian of the genome, governs the cell response, resulting in cell cycle arrest, DNA damage repair, apoptosis, and senescence. The mutational status of *TP53* has an impact on DDR, and somatic mutations in this gene represent one of the critical events in human carcinogenesis. Telomere dysfunction in cells that lack p53-mediated surveillance of genomic integrity along with the involvement of DNA repair in telomeric DNA regions leads to genomic instability. While the role of individual players (DDR, telomere homeostasis, and *TP53*) in human cancers has attracted attention for some time, there is insufficient understanding of the interactions between these pathways. Since solid cancer is a complex and multifactorial disease with considerable inter- and intra-tumor heterogeneity, we mainly dedicated this review to the interactions of DNA repair, telomere homeostasis, and *TP53* mutational status, in relation to (a) cancer risk, (b) cancer progression, and (c) cancer therapy.

## 1. Introduction

### 1.1. Genomic and Chromosomal Instabilities as Prerequisites of Solid Cancers

Genomic instability is a characteristic of most human malignancies. The disruption of genomic integrity due to the accumulation of various kinds of DNA damage, deficient DNA repair capacity, and telomere shortening constitute hallmarks of malignant diseases [1]. Defects in DNA repair increase the vulnerability of the cell to DNA-damaging agents (we have recently reviewed the sources, types, and properties [2,3,4]) and the subsequent accumulation of mutations in the genome constitutes genomic instability. Arising mutations may be either a basis of genetic diversity and natural selection during evolution or may trigger carcinogenesis. Moreover, DNA damage may also become a sequel of genotoxic stress from cellular processes such as transcription and replication, and if not properly repaired, e.g., by DNA mismatch repair, mutations are ultimately formed and accumulated [5]. Chromosomal instability, both numerical and structural, is a common form of genomic instability which can be generated via several mechanisms such as (a) incomplete or deficient repair of DNA double-strand breaks (DSBs), (b) defects in mitotic checkpoint genes, (c) critically shortened telomeres that are recognized as DSBs, and (d) as a secondary consequence of malignant transformation [6,7]. A complex cellular system called DNA damage response (DDR) prevents an accumulation of DNA damage and the subsequent formation of deleterious mutations and chromosomal damage.

### 1.2. DNA Damage Response and Its Constituents

DNA damage response (DDR) is a network of signaling pathways promoted by cells to process DNA damage. DDR is also important for both cancer development and chemotherapy outcome. DDR is a complex of events responsible for the initial detection of DNA lesions and subsequent activation of responsible signaling networks (cell cycle checkpoint induction, DNA repair, and induction of cell death). Therefore, accurate coordination of all parts of the DDR is essential to prevent malignant transformation [8].

DDR comprises multiple DNA repair pathways, DNA damage tolerance processes, and cell-cycle checkpoints that participate in a concerted way to maintain genomic integrity [8]. DDR copes with up to 70,000 altered bases per cell/day induced by diverse noxa [9]. The DDR pathway, therefore, acts as a well-coordinated, versatile, and effective system [10], closely associated with (i) DNA repair machinery, (ii) DNA replication, (iii) chromosomal segregation, and (iv) telomere maintenance [11]. DNA repair involves several further pathways such as base excision repair (BER), nucleotide excision repair (NER), mismatch repair (MMR), double-strand break repair (DSB), comprising homologous recombination (HR) and non-homologous end joining (NHEJ), and direct reversal repair (DR) (reviewed in [2]). DNA repair also comprises translesion synthesis (TLS), allowing the DNA replication past previous DNA damage and the repair of interstrand crosslinks (via Fanconi anemia genes). It should be noted that no single pathway efficiently repairs all types of DNA lesions, and some lesions serve as substrates for more than one pathway [12]. On the other hand, there are extensive interactions among the proteins of distinct DNA repair pathways [4]. DNA replication—its deregulation occurs through replication-blocking DNA lesions, the activation of certain oncogenes, the loss of function of certain tumor suppressors, and the promotion of replication stress, which triggers DSB formation and leads, consequently, to unscheduled recombination events and chromosomal rearrangements. In cancer cells, the loss of G1/S control is accompanied by p53 pathway inactivation and the suppression of cell cycle arrest and apoptosis [13,14]. Telomere homeostasis/telomere dysfunction in cells with critically short telomeres that lack p53-mediated surveillance of genomic integrity, along with the involvement of DNA repair in telomeric DNA regions, leads to genomic instability (reviewed by [15,16]).

### 1.3. TP53 Function and Its Alterations in Solid Cancers

**Figure 1:**
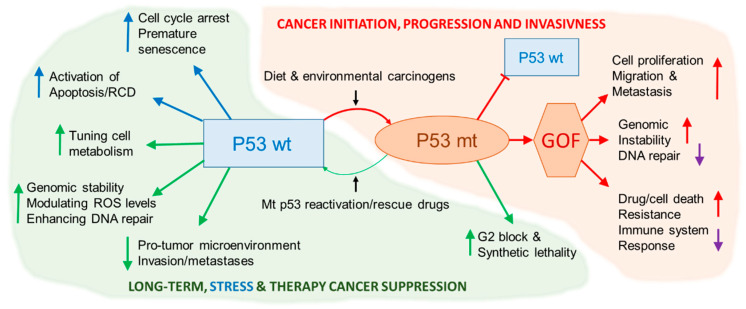
Wild-type (wt) versus mutant (mt) p53 in tumorigenesis and cancer therapy. p53 tumor suppressor maintains a long-term anti-cancer environment in normal cells by tuning cell metabolism and reactive oxygen species (ROS) levels, maintaining the genomic stability and microenvironment. It also copes with stress (e.g., DNA damage) by imposing cell cycle arrest and, eventually, cellular senescence or regulated cell death (RCD) [20]. Wt p53, via its target genes in long-term cancer prevention, regulates cellular metabolism mainly by suppressing glycolysis, enhancing DNA repair, reflecting cellular stress, modulating ROS levels, and suppressing cellular invasiveness. Cancer-associated p53 mutants, in contrast, block/attenuate wt p53 function(s) and enhance tumor initiation, progression, and invasiveness by suppressing DNA repair, immune response, and response to cancer therapy (attenuating RCD) and enhancing cell proliferation, migration, and metastases [21,22]. Mutant p53 in cancer cells can be partly rescued by mt-to-wt p53 reactivation compounds [23] or mt p53 can be exploited for therapy via synthetic lethality. Diet and environmental carcinogens represent environmental and microenvironmental (including interaction of diet with microbiota) etiological factors involved in sporadic carcinogenesis [24].

As p53 is activated in response to different types and levels of stimuli, the correct function of *TP53* is essential to protect organisms from developing cancer. As stated above, cell response induced by p53 leads to cell cycle arrest, DNA damage repair, apoptosis, and senescence. In contrast to wild-type (wt) p53, cancer-associated point mutations occur predominantly in its DNA-binding domain and the derived p53 mutant(s) (mt(s)) suppress wt p53 functions(s) and enhance tumor progression by promoting genome instability, drug and immune system resistance, and even metastatic phenotype (Figure 1). Since the study of Faeron and Vogelstein, somatic mutations in this gene represent one of the critical events in human carcinogenesis [17]. However, in our previous review on colorectal cancer (CRC), we recapitulated that the variegated phenotype of the wide spectrum of somatic mutations in *TP53*, in concurrence with the complexity of the disease, aggravates the interpretation of the mutational analysis in tumors. In addition to the somatic mutations, polymorphic features (germline variants including single nucleotide polymorphisms) of the gene may also modulate the *TP53* function [18]. There are more than 400 defined single nucleotide polymorphisms (SNPs) of this gene (http://www-p53.iarc.fr/, last update July 2020, [19]). To date, there is no possible generalization of the role of *TP53* further as a predictor of therapeutic response and prognosis. A schematic overview of the role of mutated p53 in tumorigenesis and cancer therapy is shown in Figure 1.

#### 1.3.1. The Functional Aspects of *TP53*

The mutational status of *TP53* is likely to affect DDR and wt p53-modulated processes including cellular senescence, cell cycle arrest, and regulated cell death. Wild-type (wt) p53 protein stabilization is largely dependent on its DNA damage-triggered posttranslational modifications such as phosphorylation of N-terminal serines by multiple kinases, including Ataxia Telangiectasia Mutated (ATM), Ataxia Telangiectasia and Rad3-related protein (ATR), and DNA protein kinase, and acetylation of its central DNA binding domain. Stabilized (uncoupled from MDM2-mediated proteosomal degradation and inhibition) and activated (phosphorylated and acetylated) wt p53 triggers multiple pathways and responses, including regulated cell death (RCD) and cellular senescence [25]. Activated wild-type p53 may bind to the specific DNA regions of its target genes (p21, Wip1, Noxa, and PUMA), thus mediating tumor suppressor function [26]; whereas mutated p53 in the conformation-sensitive core domain is accumulated in the cancer cells due to the inhibition of MDM2 activity by the Hsp90 complex, towards mutated p53 [27]. Though wt p53 mainly fulfills these tasks via its transcription-related activities, p53 might also, however, act in a transcription-independent manner. In the nucleus, as a transcription factor, p53 reflects the severity and persistence of DNA damage and can activate transcription of a number of RCD-inducing or -regulating genes. The most prominent wt p53-transactivated inducers of apoptosis include BH3-only proteins Puma/BBC3, and Noxa/PMAIP1; however, p53 transactivates the expression of other pro-apoptotic genes such as Bim, Bax, Apaf1, Fas/CD95, and TRAIL-R2/DR540 [28]. p53 also affects apoptosis via transcriptional repression, as in the case of the anti-apoptotic protein Bcl-2, though indirectly via increased expression of microRNA miR-34a [29]. In addition, p53 was also shown to interact with the anti-apoptotic protein Bcl-XL, blocking its ability to sequester Bax or Bak1 proteins [30]. In mitochondria, p53 was also documented to interact with the inner membrane protein cyclophilin D, enhancing mitochondrial permeability transition pore (PTP) opening and, thus, inducing PTP-dependent necrosis [31]. p53 also participates in the regulation of other modes of RCD, namely ferroptosis and autophagic cell death. p53 indirectly enhances ferroptosis by suppressing the expression of the amino acid antiporter SCL7A11 [32]. p53 feeds into autophagic cell death via the transcriptional upregulation of two of its modulators—tumor suppressor p53-inducible nuclear protein 1 (TP53INP1) and damage-regulated autophagy modulator (DRAM) [33,34]. However, p53 also actively participates in the process of cellular senescence, which is relevant in the initial stages of carcinogenesis as an anti-cancer intervention together with the activation of the adaptive immune system and T cell-mediated elimination of senescent precancerous cells [35]. The occurrence and persistence of senescent cancer or stromal cells in the advanced and metastatic stages might, in contrast, enhance cancer cell proliferation and resistance via pro-inflammatory and pro-survival cytokines produced by senescence cells (Senescence Associated Secterory Profile—SASP). p53 is directly transcriptionally responsible for the increased expression of CDK inhibitor p21/Cip1 and, thus, for the induction of cell arrest at both G1/S and G2/M checkpoints [36]. However, p21, through the induction of growth arrest/senescence, renders cancer cells more resistant to the induction of apoptosis, and the elimination of p21 expression in senescent cancer cells makes them susceptible to apoptosis [37]. In this context, wild-type p53 is a multifunctional transcription factor which is postranslationally stabilized in response to various cellular stresses, including the damaged DNA in DDR. The presence of TP53 mutations in cancer cells is generally associated with higher hypoxia-inducible factor (HIF)-1α levels. Mutated p53 probably stimulates HIF-1α stabilization in hypoxic conditions. Adaptation to hypoxic conditions is critical for tumor evolution and can contribute to angiogenesis, chemoresistance, inhibition of apoptosis, or autophagy [27].

#### 1.3.2. DNA Damage, DNA Repair, and *TP53*

Cell reaction induced by p53 clearly comprises (apart from cell cycle arrest, apoptosis, and senescence) DNA damage repair. Reactive oxygen species (ROS) represent an abundant source of DNA damage, repaired predominantly by BER [3]. Recently, Lisek et al. observed that p53R175H mutant p53 interacts with NRF2, one of the main regulators of the antioxidant response of cells. This interaction contributes to pro-survival oxidative stress response, which allows cancer cells to cope with high levels of intracellular ROS [38].

As previously demonstrated, p53 plays important roles in BER (single strand breaks repair—SSBs). Wild-type p53 protein, in contrast with that arising from missense mutations, has the ability to stimulate BER in vitro. In BER, an interaction between the bifunctional apurinic/apyrimidinic endonuclease APE1/Ref-1 and p53 was shown [39]. p53 can directly repress transcription of the APE1 gene [40]. An earlier study documented that the level of wild-type p53 correlates with BER activity in cellular extracts [41], and wt p53 can also directly enhance BER activity in vitro [42] and in vivo [43]. Furthermore, the enhancement of BER by wild-type p53 is also associated with DNA pol β [39]. The core domain of wild-type p53 maintains an intrinsic 3′-to-5′ exonuclease activity, and the p53 C-terminus participates in “sensing” and detecting DNA damage through direct binding to abnormal DNA structures. BER activity may also be modulated by p53 throughout the cell cycle as a consequence of genotoxic stress [44]. Hot-spot tumor-derived p53 mutants that do not significantly enhance BER, along with the stimulatory effect of wild-type p53 on BER, suggest their role in tumorigenesis [39]. In NER, p53 has both transcription-dependent and transcription-independent functions. It is also important for the regulation of global genome NER (GG-NER) and transcription-coupled NER (TC-NER) [45]. p53 interacts with different components of the NER pathway, including xeroderma pigmentosum, complementation group C (*XPC*), xeroderma pigmentosum type B (*XPB*), and Cockayne syndrome protein B (*CSB*) [46]. Wild-type p53 inhibits XPD (Rad3) and XPB DNA helicase activities, whereas Arg273His mutant p53 does not. Moreover, the study of Wang et al. showed that repair of UV-induced dimers is slower in heterozygote p53 mutant cells than in wt p53 human cells [47]. In vivo studies employing p53 knockout mice have demonstrated a deficiency in GG-NER, leading to an increase in chromosomal abnormalities [48]. Protein p53 also plays an important role in DSB repair (reviewed by [46]). Wild-type p53 was shown to participate in DNA repair and recombination pathways through transcriptional transactivation of DNA repair-associated genes and through an interaction with components of the repair and recombination machinery [49]. The R248W and R273H mutant forms of p53 can interact with MRE11, a part of the MRN complex in homologous recombination repair (HR). This results in the disruption of MRE11’s ability to recruit ATM to DSBs, ultimately leading to inter-chromosomal translocations due to defective ATM-dependent cell cycle checkpoints [50,51]. p53 can also interact with RAD51, which leads to interruption of RAD51 polymerization and inhibition of HR [52]. Protein association studies have also disclosed that p53 interacts with several HR components such as RPA, RAD54, BRCA1, BRCA2, BLM, and WRN (for more, see [53]). p53 has also been shown to regulate NHEJ, but its role in NHEJ is not clearly understood. Studies with XRCC4 and ligase IV in knockout mice have demonstrated that in the absence of NHEJ, DSBs remain unrepaired, ultimately leading to p53-dependent apoptosis [51]. Suboptimal or defective activity of MMR, predominantly in MLH1 and MSH2, is responsible for the hypermutation phenotype and microsatellite instability in several malignancies (CRC, ovary cancer). The functional variability of MMR may also be connected with p53. Mehigan et al. observed, in tumors with microsatellite stability (MMR-proficient), that p53 did not influence the total number of chromosomal aberrations. The decreased chromosomal aberrations were associated with the loss of hMLH1 or hMSH2 protein expressions, while p53 overexpression was not proven [54]. MMR and p53 were able to cooperate in modulating the sensitivity of the MMR-defective CRC cell line to cisplatin [55] (Table 1).

#### 1.3.3. The Function of p53 in Interaction with Telomere Homeostasis

Although only a few shorter telomeres are needed to trigger DDR, they form end-associations, leading to a DNA damage signal resulting in replicative senescence (cellular growth arrest, also called the M1 stage). In the absence of cell-cycle checkpoint pathways (e.g., p53 and/or p16/Rb), cells bypass M1 senescence and telomeres continue to shorten, eventually resulting in crisis (also called the M2 stage; [56]). In an in vitro study on breast cancer cells exposed to ionizing radiation, the authors reported p53-dependent accelerated senescence, which was associated with telomere dysfunction but not with changes in telomerase activity or telomere lengths (TLs) and expression of telomerase reverse transcriptase (hTERT) and telomerase RNA component (hTR). In the absence of functional p53, cells are unable to arrest for an extended period, resulting in apoptotic cell death, while accelerated senescence in cells expressing p53 is succeeded by proliferative recovery [57]. The loss of p53 enables a layout in which critically short telomeres are inappropriately joined to generate chromosomal end-to-end fusions. These fused chromosomes result in cycles of chromosome breakage–fusion–bridge and changes in gene copy number [58]. Importantly, due to progressing telomere dysfunction, p53 presumably activates short telomere-driven tissue failure diseases (such as pulmonary fibrosis, aplastic anemia, and liver cirrhosis). Prerequisites for cancer development are the disabling of p53 signaling (to avoid senescence) and the upregulation of telomerase to achieve cellular immortality. The discovery of activating mutations in the promoter for the telomerase reverse transcriptase gene in human cancers, along with mutations in p53 in tumors, pinpoints the importance of a telomere p53 checkpoint for malignant progression [59]. Increased p53 activity may alter genome maintenance. In mice, p53 downregulates genes that are essential for telomere metabolism (11 genes, including those encoding telomerase, helicase, and the Shelterin complex), DNA repair (such as Fancd2, a gene encoding a key protein of the Fanconi anemia DNA repair pathway which enables the completion of DNA replication by removing inter-strand crosslinks), and centromere structure. Furthermore, sustained p53 activity leads to phenotypic traits associated with dyskeratosis congenita and Fanconi anemia [60]). Ge et al. discovered that a high load of intrinsic DNA damage is present in cancer cells counteracting telomere shortening by alternative telomere lengthening (ALT). This leads to stress resolved by activating a p53-independent, c-Jun N-terminal kinase—JNK/c-Myc-dependent apoptotic pathway. The authors concluded that p53 and AKT may act as key factors that suppress spontaneous apoptosis in ALT cells [61]. Notably, ALT cells expressing wild-type p53 show much lower apoptosis than p53-deficient ALT cells. Certainly, the inhibition of p53 or AKT selectively induces the rapid death of ALT cells in vitro, and the p53 inhibitor severely suppresses the growth of ALT cell xenograft tumors in mice. These findings reveal a previously unrecognized function of p53 in suppressing apoptosis [61].

A better comprehension of the crosstalk between DNA repair (and key repair proteins), telomere homeostasis, the role of p53, and apoptosis unambiguously emerges as prerequisite in tracking carcinogenesis, and we provide, here, a brief survey of (a) cancer risk, (b) cancer progression, and (c) treatment prognosis. Although the *TP53* function may be modulated on various levels, i.e., on the genetic (mutations, variants), transcriptional (splacing sites, epigenetic silencing/miRNA interference), or protein level, we mainly focused on the former aspect of TP53.

## 2. The Impact of Interactions between Altered p53 and DNA Repair on Cancer Risk

### 2.1. Most Frequent Mutations in Tumorigenesis

The p53 protein is mutated in about 50% of human cancers and inactivated (nuclear exclusion or MDM2 overexpression) in approximately an additional 20% of human tumors. More precisely, almost one third of all cancer types exhibit a *TP53* mutation rate exceeding 50% and more than one half have this rate higher than 30%. The highest *TP53* mutation rates have been recorded in uterine carcinosarcoma (over 91%) and ovarian serous adenocarcinoma (83%) Apart from losing its tumor-suppressive activities, mutant p53 may acquire pro-oncogenic activity [21]. There are germline mutations in *TP53* (missense mutations account for 74% of *TP53* mutations, nonsense mutations for ∼9%, and splice mutations for ∼8%). The majority of mutations occur in the highly conserved DNA-binding domain and the six most common “hot spot” mutations are found in codons 175, 245, 248 (two common substitutions), 273, and 282 (https://p53.iarc.fr, last update July 2019; [19]) and are associated with Li–Fraumeni syndrome, a hereditary cancer syndrome [62]. *TP53* mutations comprise missense, nonsense, frameshift insertions or deletions, silent, and splice-site mutations. The most frequent classes of *TP53* mutations are missense (accounting for 62%), nonsense (14%), and frameshift deletions (9%; for review, see [63]). *TP53* is a tumor suppressor gene, and most of the somatic mutations are missense-type at “hot spots” [64]. *TP53* mutations occur as “gain of function” (GOF) or “loss of function” (LOF) mutations. GOF mutations trigger the accumulation of mutant p53 in the affected cells, suggesting the oncogenic role of mutant p53. On the other hand, no detectable p53 in tumor cells indicates the presence of an LOF mutation in the *TP53* gene [65]. There are two different mechanisms of *TP53* alterations: the former comprises *TP53* gene deletions (induced by transacting mutation, deletion, insertion, and frameshift substitution), and the latter are *TP53* missense mutations [66]. *TP53* mutations mostly occur in the central DNA-binding domain of p53 and result in an inactivated transcription factor function [67], loss of tumor suppressor activity, or gain of function mutation. Around 40% of cancer cases with p53 mutants keep a wild-type *TP53* allele; however, a p53 mutant can affect the role of the remaining wild-type p53 by heterodimerization and blocking its wt-linked functions [64]. For missense, *TP53* mutations are typical of the concomitant deletion of the other allele through the deletion of chromosome 17p [68]. The emerging loss of heterozygosity results in an abrogated tumor suppressor function of the affected allele [66]. Common missense mutations disrupt the ability of p53 protein to bind DNA and transactivate target genes [69]. Highly frequent hot spot missense mutations are a key feature of GOF. GOF mutations are characterized by a loss of sequence-specific DNA binding and they revert the p53 function from tumor suppressor to oncogene [70], ultimately promoting cell transformation, tumor progression, metastasis, and chemoresistance. GOF p53 mutant can bind to a number of genes and induce their expression—for instance, epidermal growth factor receptor (*EGF*), *Bcl-XL*, *c-Myc*, *FosE2F1*, *NF-Kb*, *SMADs*, and *MDR-19* [71]. Different GOF p53 mutants also have a distinct binding affinity to different transcription factors. For example, the mutant R248W binds to *MRE11*, whereas R273H binds to *NF-Y*, *MRE11*, or *YAP1*. The p53 mutants R175H, R248W, R273H, Y220C, E258V, R110P, and R282W can interact with p63 and p73. Moreover, p53 mutants may exhibit increased chromatin accessibility and enhance gene expression within the spans of accessible chromatin [66].

### 2.2. Studies on the Interactions of DNA Repair, Telomere Homeostasis, and p53 Mutational Status in Cancer Predisposition

An effect of somatic *TP53* mutations in tumorigenesis may be illustrated during the transition from benign colonic adenomatous polyps to sporadic CRC [72]. While the former exhibits frequencies ranging between 15% and 30% [73], in the latter, the frequencies of *TP53* mutations are considerably higher, accounting for 34% of mutations in proximal colon tumors and 45% in distal colorectal tumors [74]. An earlier study on CRC patients documented an association of *TP53* mutations with the tumor site and adjuvant treatment, suggesting prognostic significance of this genetic alteration [75]. Regarding CRC, a recent review reported that mutations in *TP53*, *KRAS*, *APC*, and *PIK3CA,* occuring in left-sided tumors, demonstrate polypoid-like morphology and are associated with a chromosomal instability pathway [76,77].

In an elegant study on volunteers over time, the authors sought for associations between p53 activity and measured UV-induced DNA damage via cyclobutane pyrimidine dimers (CPDs), since p53-mediated excision repair of UV-induced DNA damage is a critical effector in xeroderma pigmentosum (XP) and in photoproduct-related skin cancer. The authors found a statistically significant decrease in p53 expression and CPD levels [78].

Barrett esophagus (gastrointestinal precancerosis) evolves on a background of chronic inflammation linked to free radical formation and oxidative DNA damage formation. In a study aiming to evaluate the link among 8-hydroxydeoxyguanosine, *OGG1* polymorphism, telomerase activity, telomere length, and p53 mutation in Barrett progression, the authors concluded that disease progression is associated with an accumulation of oxidative DNA damage causing telomere instability, telomerase activation, and, at a late phase, with mutations in the p53 gene. This invalidates its role as the checkpoint of proliferation and apoptosis and paves the way for cancer progression [79].

### 2.3. Studies on the Interactions of DNA Repair, Telomere Homeostasis, and p53 Mutational Status in Cancer Risk

In a study employing a set of serological markers for telomere dysfunction, the authors suggested that DNA damage (by decrease in SOD) and tissue alteration of p53 (a significant increase) may be associated with gastrointestinal (GI) tumors. There was a significant correlation between biomakers of telomere dysfunction, p53, oxidative stress, and the malignant stages of GI cancer patients [80]. An interesting study has recently been undertaken to investigate the association of telomeric alterations (assayed for in-blood leucocytes) with the risk of pediatric solid tumors on the background of *TP53* rs1042522, *MDM2* rs2279744, and *CDKN1A* (*p21^cip1^*) rs1059234 single nucleotide polymorphisms (SNPs). The results suggested that children with longer TLs were at increased risk of developing solid tumors. The risk for tumors increased nearly threefold for the interaction of TL, *TP53* rs1042522, and *p21^cip1^* rs1059234; however, the interaction effect was less than additive. On the contrary, *MDM2* rs2279744 GG genotype significantly reduced the pediatric solid tumor risk [81]. Scalis et al. investigated colorectal adenoma and adenocarcinoma to address the association between oxidative DNA damage and mutations in *TP53* gene. The authors observed an enhanced *TP53* level in non-malignant tissues from patients with cancer, as compared to mucosa from healthy individuals. By comparing tumor tissues with non-malignant tissues, the former exhibited higher *TP53* levels. Likewise, tumor tissues had significantly higher oxidative DNA damage levels than non-malignant tissues. In all of the samples, the authors recorded the link between oxidative DNA damage and *TP53* mutation. The authors concluded that oxidative DNA damage along with *TP53* mutation are important contributors to colorectal adenoma–carcinoma transition [82]. Apart from somatic mutations in the gene, polymorphic features (germline variants including single nucleotide polymorphisms) of the gene may also modulate its function [83]. Wang et al. investigated germline variants in *ATR/CHEK1* and *ATM/CHEK2* (rs35514263 in *ATR*; rs492510, rs558351 in *CHKE1*; rs189037 in *ATM*; rs2236141, rs5762748, rs2236142, and rs9620817 in *CHEK2*) acting in DDR and their association with CRC risk in a Chinese population. Individuals bearing the rs189037 A allele were at significantly higher risk of CRC compared to those carrying the G allele. The risk was more pronounced in older patients and those with rectal cancer, early stage and higher grade. A bioinformatic analysis suggested that rs189037 may change the secondary structure of the protein [84].

## 3. Studies on the Interactions of DNA Repair, Telomere Homeostasis, and p53 Mutational Status in Cancer Progression and Prognosis

Since mutations in *TP53* tumor suppressor might lead to dramatic phenotypic changes and diversification of cell responses to stress, the authors analyzed the functional fingerprints of sporadic breast cancer-related p53 mutants, many of which are also associated with familial cancer proneness such as the Li–Fraumeni syndrome and germline *BRCA1/2* mutant-associated cancers, and concluded that functional and non-functional missense mutations may affect the clinical behavior and outcome of breast cancer patients [85]. However, regarding tumor progression, the area of p53 mutations is complex by itself and requires cautious interpretation. As an example, Siegel et al. addressed early drivers in breast cancer metastasis and identified tumor-specific drivers by integrating a known protein–protein network in formation with RNA expression and somatic DNA alterations. Additionally, genetic drivers were predominantly established in the primary tumor and maintained through metastatic spreading. The authors revealed that most genetic drivers were DNA copy number changes; the *TP53* mutation was a recurrent founding mutation regardless of subtype. In metastasis, somatic mutations in the estrogen and androgen receptor genes were identified as unique genetic drives. These results highlight the complexity of metastatic spreading, regardless of the fact that knowledge on the role of DNA repair and telomere homeostasis remains rudimentary [86]. The protein expression of cell cycle regulators and p53 have been analyzed for their prognostic role in various breast cancer subtypes. The authors documented that the expression of cell cycle regulators in the absence of p53 protein is associated with a favorable prognosis in operable breast cancer [87]. The prognostic value of *TP53* mutations has been investigated in metastatic CRC patients who underwent surgical resection of hepatic metastases (HMs). Patients with a very poor prognosis were defined by the concurrence of equal or more than three HMs and *TP53* mutations, whereas immunohistochemical analysis of p53 and p21 showed no association with survival. In CRC patients undergoing surgical resection of HMs, *TP53* mutational status may pose an important prognostic factor [88]. In a meta-analysis, the authors demonstrated that *KRAS* mutations along with p53 mutations were associated with CRC metastasis, including lymphatic and distant metastases. Moreover, CRC patients with a KRAS mutation, p53 mutation, or SMAD4 mutation were at an increased risk of distant metastasis [89].

The above studies did not, however, address DDR and/or telomere homeostasis. An additional study has addressed the prognostic relevance of *TP53* mutations (at codons 273, 248, 175, 282, and 245), MMR deficiency (measured as microsatellite instability status—MSI), and *KRAS* mutation status in a group of patients with stage III CRC, randomly assigned for adjuvant treatment with fluorouracil-based chemotherapy. The authors concluded that both mutant *TP53* and MSI-H seemed to be prognostic indicators for disease-free survival (DFS), but only *TP53* retained statistical significance after adjusting for clinical heterogeneity. Thus, in patients with stage III colon cancer, treated with adjuvant therapy, the presence or absence of a *TP53* mutation should be considered as a preferable predictor for DFS than MSI status [90]. However, a concurrence of both, MMR deficiency with *TP53* mutations, was only detected in nine patients out of 44 diagnosed as MMR-deficient from total 391 cancer patients included in the study. This concurrence occurred in about 20% of MSI-H patients and further, more extensive studies should analyze possible interactions [85]. In a study conducted on non-small cell lung cancer (NSCLC) patients, the authors disclosed that loss of heterozygosity (LOH) for hDMP1 is connected with better prognosis, whereas that of *TP53*, with impaired prognosis [91]. Regarding lung adenocarcinoma, the authors reported poor prognosis in patients bearing mutated *TP53*. However, *TP53* and *EGFR* co-mutation exhibited an even worse impact on patient prognosis [92]. A 17.5-year follow-up study investigated the prognostic value of circulating Bcl-2 and anti-p53 antibodies (p53Abs) in breast cancer patients. The authors demonstrated that high serum levels of p53Abs and presence of Bcl-2 predispose patients to unfavorable prognosis [93]. In a strong study comprising 694 gastric cancer patients, the authors observed that aberrant p53 expression (by immunohistochemistry) was associated with worse prognosis. The prognosis seemed to differ in relation with tumor localization [94].

A study postulated that the tumor suppressor protein p53 deficiency or inactivation can lead to the activation of telomerase enzyme, with subsequent consequences on the ability to detect DNA damage connected with apoptosis. An interaction between p53 and telomere homeostasis was demonstrated, for instance, by using bioactive compounds such as phenolic compounds, saponins, and alkaloids. These compounds modulated p53 expression and indirectly affected the TL. The main concept of the report was that phenolic compounds, saponins, and alkaloids presumably interfere with cancer progression by stimulating p53 expression with subsequent pro-apoptotic onset. Additionally, the anti-apoptotic activity was suppressed and telomerase activity was protected [95]. In our recent study, we demonstrated that *Ganoderma lucidum* (GLC) extract induced oxidative DNA damage selectively in CRC cell lines (HCT116, HT29, and HCT116^p53-/-^), whereas it protected non-malignant cells from the accumulation of reactive oxygen species. The accumulation of DNA damage caused sensitization of cancer cells to 5-Fluorouracil (5FU), resulting in the improved anti-cancer effect of 5FU. We also observed increased levels of p53 protein following GLC treatment and hypothesized that GLC can restore the tumor suppressor function of p53. This may lead to restoration of cell cycle regulation and cell cycle arrest and apoptosis. Although p53 in general is not necessary for the induction of DNA damage, it is critical for the occurrence of cell death. In the presence of p53, GLC dramatically enhanced the cytotoxicity of 5FU by initiating oxidative DNA damage, specifically in colorectal cancer cells [96]. These interesting interactions applicable in cancer cell biology clearly warrant further extensive investigation.

## 4. Studies on the Interactions of DNA Repair, Telomere Homeostasis, and p53 Mutational Status in Modulating Cancer Therapy

### 4.1. Interactions of DNA Repair, Telomere Homeostasis, and p53 and Their Mechanistic Impact (Underlying Mechanisms In) on Cancer Therapy

Mutant p53 also plays an important role in cancer resistance. Moreover, in CRC patients with mutant p53, chemoresistance is present in most cases and these patients have a poorer prognosis than those with wild-type p53 [97]. Mutant p53 (D281G) has been shown to induce expression of the *MDR1* gene (which is suppressed by wild-type p53 in normal conditions), which encodes P-glycoprotein, an energy-dependent drug efflux pump [98,99]. P-glycoprotein is an important player in cancer chemoresistance development.

DDR, along with ATR-Chk2 signaling in p53 activation during cisplatin-induced apoptosis, represents a classical study on the mechanisms underlying the therapeutic effects of cisplatin in cancers. The authors demonstrated an early DNA damage response during cisplatin treatment of renal cells and tissues. In DDR, a critical role for ATR, but not ATM or DNA-dependent protein kinase (DNA-PK), in cisplatin-induced p53 activation and apoptosis was reported. The authors documented that ATR is specifically activated during cisplatin treatment and co-localizes with γH2AX, forming nuclear foci at the site of DNA damage. Downstream of ATR, both Chk1 and Chk2 are phosphorylated during cisplatin treatment in an ATR-dependent manner. In vivo in C57BL/6 mice, ATR and Chk2 are activated in renal tissues following cisplatin treatment. Overall, the results suggest an important role for DDR mediated by ATR-Chk2 in p53 activation and renal cell apoptosis during cisplatin nephrotoxicity [100]. Furthermore, it was documented that MMR plays a role in DDR via key signaling kinases, ATM and Rad3-related ATR, in response to various types of DNA damage (including those generated by chemotherapy [101]. As stated above, ATR plays an essential role in the repair of replication-associated DNA damage, while ATM is activated by DNA double-strand breaks. The advent of ATM/ATR inhibitors (ATMi, ATRi) and their implementation in cancer monotherapy and combinational therapy are analyzed along with p53, ATM, ARID1A, and other DDR aberrations as markers of treatment response [102]. For instance, Williamson et al. documented both in vitro and in vivo that defects in ARID1A sensitized tumor cells towards ATR. Once tumor cells possess ARID1A mutations, subsequent ATR inhibition initiates premature mitotic entry, genomic instability, and apoptosis [103].

However, in the mechanistic assessment of DDR, p53, and telomere homeostasis in cancer treatment, the character and type of DNA damage and efficiency of DNA repair capacity have to be adequately considered. Regarding p53 and drug resistance, the modes of action of anti-cancer drugs, including the type of DNA damage induced, warrant further investigation both in vitro and in vivo.

### 4.2. The Interactions of DNA Repair, Telomere Homeostasis, and p53, and Their Underlying Mechanisms in the Resistance towards Cancer Therapy

ATM, a core component of the DNA repair system, is activated to enhance the homologous recombination (HR) repair pathway upon DNA double-strand breaks. ATM inhibitors such as KU-55933, KU-60019, KU59403, CP-466722, AZ31, AZ32, AZD0156, and AZD1390 have been investigated for their antitumor effects, and increased cancer cell sensitivity to radiotherapy following ATM inhibition was recorded. Moreover, combination with poly(ADP-ribose)polymerase (PARP) or ATR inhibitors is a basis synthetic lethality in some cancers [104]. Interestingly, ATRi inhibitors act more effectively with genotoxins in cells with defects in the p53 pathway than in those that are p53-proficient. This is likely due to the loss of the G1 check point and/or to relaxed S-phase entry [105].

Radiation therapy is among the current treatment modalities for many cancers, including CRC. Recent studies have suggested that the presence of cancer stem-like cells (CSCs) may be responsible for therapeutic resistance and disease relapse. By using HCT116 and HCT15 cells and derived colonospheres, the authors recorded a more pronounced cell survival, decreased γH2AX foci and SSBs, and increased ERCC1 and p-ATM expression in colonospheres post-irradiation than in colonospheres with efficient DDR. Differential expression of a developmental marker, CSC markers, and telomeric components was observed after irradiation. The authors documented the significance of CSC phenotype in colonospheres with increased DNA repair capacity and differential expression of CSC markers and telomeric components as a reason for CSC radioresistance [106].

### 4.3. The Interactions of DNA Repair, Telomere Homeostasis, and p53 Utilized in Cancer Therapy

ATM and ATR kinases, which induce cell cycle arrest and facilitate DNA repair via their downstream targets, are inseparable players in DDR. The inhibition of DDR emerges as a promising therapeutic strategy in cancer therapy since (i) resistance to genotoxic therapies has been associated with increased DDR signaling and (ii) many cancers have defects in certain components of the DDR. This increases their dependence on the remaining DDR pathways for survival. Highly selective small-molecule inhibitors of ATM and ATR are currently being tested in preclinical and clinical studies. The use of these inhibitors both in combination with radio- or chemotherapy and in synthetic lethal approaches shows a promising approach to treat tumors with deficiencies in DDR components [107]. An additional report summarized the status of the development of ATR and CHK1 inhibitors and mechanisms, by which ATR and CHK1 inhibition trigger cell killing in association with exogenous DNA-damaging agents [108]. Preclinical data have, so far, demonstrated that ATR inhibition in specific molecular contexts evokes synthetic lethality, and it exhibits synergy in combination with different antitumor therapies (such as chemotherapy, radiotherapy, and poly(ADP-ribose)polymerase (PARP) inhibitors [102]. Synthetic lethality combining the inhibition of DNA repair with the inactivation of S or G2 checkpoints might be a suitable strategy for the treatment of cancer cells/tumors containing mutant p53. As these cells, which are due to wt p53 inactivation, lack the p21/Cip1/Waf1-induced G1 checkpoint, the simultaneous DNA damage and compromizing G2 checkpoint leads to their effective elimination by cell death signaling. The treatment of triple-negative breast cancer (TNBC) containing mutant p53 with the pan-CDK inhibitor roscovitine followed by doxorubicin has led to increased overall survival of mice with implanted TNBC tumors [109]. Deletion of the DNA repair protein XPA in lung adenocarcinomas enhances the synthetic lethality between cell cycle kinase MK2 inhibitors and cisplatin in cells with mutant p53 [110]. Interestingly, inhibition of the G2 kinase WEE1 using the specific drug AZD1775 enhances carboplatin efficacy in ovarian cancer cells with mutant p53 and in phase II trial patients with ovarian cancer [111]. Currently, there are over 20 clinical studies exploiting WEE1 kinase inhibitor AZD1775 in cancer treatment. To name a few examples, a two-stage single arm phase II study was conducted to assess the activity of the Wee1 inhibitor AZD1775 as a monotherapy in recurrent uterine serous carcinoma (USC). This study demonstrates promising clinical activity of AZD1775 monotherapy in women with USC [112]. A Randomized Phase II Trial of AZD1775 plus paclitaxel and carboplatin for women with platinum-sensitive *TP53*-mutant ovarian cancer showed improved progression-free survival. There were also observed clinical benefits for patients with different *TP53* mutation subtypes and possible response biomarkers were observed [113]. The concept of synthetic lethality has often been applied in breast and ovarian cancer patients with mutated *BRCA1/2*. Specifically, administration of the PARP inhibitor olaparib improved progression-free survival in women with high-grade serous ovarian cancer (HGSOC). Sensitivity to olaparib correlated with a complete response to chemotherapy (CT) in women harboring *TP53*, *BRCA1*, and *BRCA2* mutations. The authors concluded that a greater response to olaparib is rather multifactorial and associated with homologous recombination repair deficiency (e.g., in *BRCA1/2*; [114]). The above strategies aimed at DDR as a therapeutic target in ovarian cancer treatment have recently been reviewed [4].

In the recent study of Wilsker et al., the authors investigated the pharmacodynamic activation of the DDR pathway in tumors following anti-cancer treatment. They assessed the activation of three protein biomarkers reflecting DNA damage recognition and repair (γH2AX, pS343-Nbs1, and Rad51) simultaneously in a quantitative multiplex immunofluorescence assay (IFA) in tumor tissues following exposure to DNA-damaging agents. Baseline DDR biomarker levels in a CRC microarray as well as those in xenograft-bearing mice and clinical CRC biopsies from subjects exposed to DNA-damaging therapeutic regimens exhibited marked intratumor heterogeneity, partially explainable by the cell-cycle dependency of DNA damage biomarker expression [115]. Muscle-invasive bladder cancer (MIBC) is neoadjuvantly treated with cisplatin-based chemotherapy; however, there are limitations posed by the toxicity. The authors investigated the suitability of three cycles of neoadjuvant accelerated methotrexate, vinblastine, doxorubicin, and cisplatin (AMVAC) therapy and correlated p53 mutation status and TL with response and toxicity. The authors summarized that treatment with AMVAC diminished the toxicity, yet TL and p53 mutation did not predict either response or toxicity [116]. The study comprehensively addressed the transcriptional downregulation of many cell cycle genes as a pivotal mechanism resulting in p53-mediated cell cycle arrest. Apparently, the p53-p21-DREAM-E2F/CHR pathway (p53-DREAM pathway, involving genes encoding DNA repair, telomere homeostasis, and Fanconi anemia) governs p53-dependent repression of the cell cycle. This pathway executes control over more than 250 mostly cell cycle-associated genes, spanning from the G1 phase to the end of mitosis. The p53-DREAM pathway is involved in the control of all checkpoints from DNA synthesis to cytokinesis, including G1/S, G2/M, and spindle assembly checkpoints; therefore, defects in the p53-DREAM pathway contribute to a loss of checkpoint control and promote chromosomal instability and cellular aneuploidy. CDK inhibitor drugs such as palbociclib, abemaciclib, and ribociclib emerge as targets in cancer treatment when DREAM function is abrogated [117].

## 5. Open Issues and Perspectives

The appearance of the DREAM pathway provides a clear example of the complexity of the interplay of DDR, telomere homeostasis, and p53. Recent investigations clearly indicate the necessity to disregard simple solutions and concepts. Cancer, as a complex, multifactorial disease with pronounced intrinsic heterogeneity, may not be comprehended or cured on the basis of a single pathway/target. Interactions between important and relevant pathways and their interphase and transitions between biomarkers are gradually gaining attention and, once this complexity is understood, will give optimism for individualized, effective cancer therapy.

### 5.1. The Interactions of DNA Repair, Telomere Homeostasis, and p53 in the Context of Epigenetic Regulations

As stated above, DNA repair represents a barrier against genotoxic stress causing metabolic changes, inflammation, and cancer. The expression level of DNA repair genes determines the resulting DNA repair capacity. The expression levels (not only in DNA repair genes, but in general) are modulated by mutations in their coding or promoter regions, changes in the expression of transcription factors activating or repressing the genes, and/or epigenetic factors such as non-coding RNAs, histone modifications, and CpG promoter methylation or demethylation levels. Christmann and Kaina, in their review, elucidated the role of DDR components (p53, PARP-1, and GADD45a) in the regulation of DNA (cytosine-5)-methyltransferase DNMT1, the key enzyme responsible for gene silencing. The authors also stressed the importance of the epigenetic silencing of DNA repair genes for tumor formation and its therapy. They addressed the issue of whether genotoxic stress itself can lead to epigenetic alterations of genes, encoding proteins involved in the defense against genotoxic stress [118]. In the case of CRC, very few DNA repair genes have been silenced by hypermethylation [119,120]. In contrast, hypermethylation of the *MLH1* gene underlies around 10% of all sporadic CRC. The role of non-coding RNA binding in 3′-untranslated regions of DNA repair genes has been reported by us [121,122,123,124]. In our recent report, we have highlighted, for the role of DNA repair in ovarian cancer, the necessity to investigate interesting links between DNA damage, DNA repair, and DNA methylation/demethylation and advocated for further studies on the epigenetic regulation of DNA repair/DDR via non-coding RNAs in relation to disease onset, prognosis, and therapy outcome [4].

### 5.2. The Interactions of DNA Repair, Telomere Homeostasis, and p53 in the Context of the Immune System

The role of immune surveillance in human cancer has been extensively investigated in the past decade. This applies to immune cells (and other cells, such as endothelial cells and stromal fibroblasts) and the tumor microenvironment. It has been postulated that carcinogenesis shares similar features with chronic inflammatory processes. The inferred terms of “hot” or “cold” tumors refer to the signs of inflammation (hot) when a tumor has already been infiltrated by T cells (e.g., MSI-high CRC), and this usually correlates with a response to immunotherapy. These processes are associated with the involvement of DNA repair, telomere homeostasis, and p53. Involvement of an evolutionarily conserved cellular response pathway DDR in the maintenance of genome integrity in epithelial stem cells was recently reported by Gronke et al. In intestinal epithelial stem cells, cytokine interleukin-22 (IL-22), produced by group 3 innate lymphoid cells (ILC3) and γδ T cells, takes part in DDR regulation. The authors demonstrated, on a mouse model, that following DNA damage, DDR is effectively initiated by IL-22. Stem cells that were exposed to carcinogens and deprived of IL-22 signals evaded DDR-directed apoptosis, accumulated mutations, and were prone to colon cancer onset [125]. Recent investigations indicate crosstalk between DNA repair machinery and the immune system. In the report of Mukherjee et al., the authors addressed ATM, BRCA1, DNA-PK, FANCA/D2, MRE11, MUS81, NBS1, RAD51, and TREX1 and their role in suppressing cytosolic DDR-mediated immune signaling. Interestingly, the immune system exhibited improper signaling in the presence of defects in DNA repair pathways, suggesting that most DDR factors negatively impact the immune system. Apart from the roles of DDR in DNA repair and replication, this system may prevent inappropriate activation of immune signaling. The authors concluded that sound knowledge of the mechanisms by which different DDR factors function in immune signaling is essential for preventing autoimmunity, cellular senescence, and cancer [126].

### 5.3. The Interactions of DNA Repair, Telomere Homeostasis, and p53 and the New Concept in Cancer Therapy

The study by Lin et al. on mitochondrial DNA (mtDNA) copy number represents an interesting attempt to investigate extragenomic DNA status to understand the biology and the progression of cancer, including its therapy [55]. Recent findings have illustrated that cancer cells with dysfunctional mtDNA (using cells devoid of mtDNA, ^0^ cells), when grafted in mice, acquire mtDNA from the host by “importing” whole mitochondria [127]. The reason for the import of mitochondria from the stroma to ^0^ cells is linked to the restoration of mitochondrial respiration, a pre-requisite for tumor formation. However, there is virtually no information on the conformity between genomic and mtDNA, on the role of DNA damage and its repair in mtDNA, and on how the changes in mitochondria and mtDNA are reflected in DDR and telomere homeostasis. This insufficient knowledge applies to cancer cells and their microenvironmental context and, particularly, for any therapeutic intervention.

## 6. Conclusions

By reviewing the current knowledge on the interactions of DNA repair, telomere homeostasis, and p53 mutational status in solid cancers in terms of risk, prognosis, and prediction, we encountered the following issues: (i) There is a scarcity of data on these interactions, even though all of the players are unequivocally considered as individually important players in cancer onset, prognosis, and treatment (every DDR process is functionally impaired to some extent in one or more cancer types, *TP53* is mutated in many cancers, and telomere homeostasis is also disturbed in the majority of cancers); (ii) the ability of cancer cells to undergo apoptosis in response to DNA-damaging agents seems to be substantially influenced by the cell type of origin, and these intrinsic differences between cell types may also affect therapeutic response; (iii) there is a scarcity of data on TP53, DNA repair capacity, and telomere homeostasis in combination in cancer onset, prognosis, and treatment; (iv) mutant p53 induces resistance in solid tumors to currently used chemotherapeutics, and therefore, exploitation of the properties of mutated *TP53* (e.g., lost ability to activate cell cycle arrest) may be useful for potential novel treatment strategies focusing, e.g., on synthetic lethality; (v) inherited genetics of *TP53*, telomerase reverse transcriptase (*TERT*), and the telomerase RNA component (*TERC*) genes and DDR pathways could be utilized to further define patient populations in their abilities to respond to either DNA damage or p53-targeted therapies; however, genetic variants must be functionally characterized and epistasis should be taken into consideration; (vi) the potential utilization of telomere homeostasis in cancer therapy has been suggested and has to be further expanded; (vii) in assessing the role of DDR, p53, and telomere homeostasis in cancer treatment, the character and type of DNA damage and the efficiency of DNA repair capacity have to be adequately considered; (viii) by assessing these important pathways, their epigenetic regulation should not be neglected.

## Figures and Tables

**Table 1:** Effect of *TP53* on different DNA repair pathways; BER—base excision repair; NER—nucleotide excision repair; HR—homologous recombination; wt—wild-type.

*TP53* Status	DNA Repair Pathway	Effect on DNA Repair	Citation
*TP53* missense mutation	BER	APE1 regulation	[41]
Hot-spot tumor-derived *TP53* mutants	BER	Does not enhance activity of repair pathway in comparison with wt p53	[39]
Arg273His mutant *TP53*	NER	Does not inhibit XPD (Rad3) and XPB DNA helicase activities in comparison with wt p53	[47]
p53 knock out	NER	Increase in chromosomal abnormalities	[48]
R248W and R273H mutant *TP53*	HR	Interaction with MRE11; inter-chromosomal translocations	[50]

## Data Availability

The review article is based on literature search, it is not an original research article.
